# The developments in amniotic membrane transplantation in glaucoma and vitreoretinal procedures

**DOI:** 10.1007/s10792-022-02570-5

**Published:** 2023-01-30

**Authors:** Rohit Sharma, Vivian Nappi, Theodoros Empeslidis

**Affiliations:** 1 Eye Department, University Hospitals Derby & Burton NHS trust, Burton, UK; 2 School of Medicine, University of Nottingham, Nottingham, UK; 3 Ophthalmology Department, Nottingham University Hospital NHS Trust, Nottingham, UK; 4 Ophthalmology Department, Ashford Hospital London, London, UK

**Keywords:** Amniotic membrane, Trabeculectomy, Glaucoma treatment, Vitreoretinal procedures, Macular hole, Retinal detachment

## Abstract

The main reasons why Amniotic Membrane (AM) is transplanted in Ophthalmology are: to provide a substrate for cellular growth and to provide tectonic support or as a biological bandage and barrier that protects the wound to facilitate an environment for wound healing. The application of AM is well-documented in corneal disorders of various aetiologies [1], however, research within the field has highlighted how it can be used in conjunctival disorders and most recently, in glaucoma and vitreoretinal procedures. This review explores the preservation modalities of AM and summarises the current literature regarding AM transplantation in Glaucoma and Vitreoretinal conditions. AM transplantation in conjunction with trabeculectomy was reported to be used in two different surgical techniques. They differ in relation to the position of the implant: below the scleral flap or over the entire exposed sclera. The results of these studies suggest that AM transplant is a safe procedure that helps in the improvement of the intraocular pressure when associated with trabeculectomies. Moreover, it enhances trabeculectomies success rates when used along with mitomycin C [2]. The use of AM is also described for managing leaking blebs. It is mentioned to be a suitable alternative to conjunctival advancement. Regarding AM transplantation in glaucoma shunt or valve surgeries, the current literature is relatively limited. However, AM has been described as a good tectonic support for shunt procedures [3]. Successful results are described in the literature for surgical treatments using AM plug for vitreoretinal procedures. In particular macular hole closure and rhegmatogenous retinal detachment. In conclusion, AM transplant is a very promising and versatile adjutant therapy. However, further studies are also required for a better understanding and refinement of surgical techniques.

## Introduction to amniotic membrane transplantation

### Historical development of amniotic membrane transplantation

AM is the innermost layer of the placental sac, which nurtures the baby during pregnancy [4]. The AM was first transplanted in ophthalmology in the 1940’s by De Rotth et al. [5], with only fresh AM being available as a treatment option. However, AM transplantation gained popularity in the 1990’s as a widespread and established treatment once preservation modalities were developed to store and transport tissue.

The use of AM in corneal and ocular surface disease pathologies is already well documented in the literature. So it will not be explored in detail in this paper. AM is known for example, to be helpful in the treatment of dry eye disease [6] and ocular burns [7] among others. AM is transplanted in different forms for ocular surface conditions such as patch, graft [7], extract or extract eye drops [8].

### Amniotic membrane structure

Different descriptions of the AM layers are found in the literature. To the best of our knowledge, the AM has been commonly subdivided into 3 layers [9–11]: (i) epithelium, a monolayer of cuboidal cells which are metabolically active; (ii) basement membrane (iii) and stroma layer, an avascular layer which itself can be subdivided in three other layers (a compact layer, a fibroblast layer and a spongy layer).

However, a different classification (into 5 layers) has also been found in the literature. Walkden [4] describes the layer structure of the AM as: (i) epithelium, (ii) basement membrane, (iii) stromal extracellular matrix (ECM) - which consists of compact and fibroblast layers. (iv) spongy layer and (v) Chorion.

### Methods of preservation

To allow for the ease of access to tissue transplantation, AM preservation methods have been developed to provide the ability to have the therapy thoroughly tested for infectious diseases, transported, and stored prior to use. AM is collected from consenting mothers undergoing elective caesarean section, processed in aseptic conditions, and tested for a range of transmissible diseases prior to preservation [4, 12]. The aim of AM preservation is to keep it as close to the native tissue as possible and to provide an on-demand, accessible therapy.

#### Cryopreservation

Cryopreservation involves freezing the AM at −80 °C, after the tissue has been washed with a cryoprotectant such as phosphate buffered silane in dimethylsulfoxide [13] or Eagle’s Minimum Essential Medium [14]. This form of preservation accounts for a largest amount of AM products on the market [15].The degradation of the tissue through enzymatic reactions has been reported to be limited at this temperature and the AM is deemed non-viable. Tissue must be kept frozen during transport and storage, meaning it is subject to the challenges associated with cold chain logistics and prior to use it must be thawed. Literature has noted that this thawing process causes a loss in the soluble factors that are presumed to be beneficial for healing [15–18]. Additional to this, ice crystal formation during the freezing process has been reported to cause issues to the cellular integrity and tissue structure [15].

#### Freeze-drying (or lyophilisation)

Freeze drying of AM conventionally involves the same initial freezing stages as cryopreservation, as the tissue is frozen to −80 °C and sublimation is used to remove water from the tissue. This sublimation process halts any enzymatic reactions taking place within the cells that can alter the tissue state [19]. This presents a dry stable tissue, which does not require cold chain storage and logistics that can be rehydrated prior to use [20]. However, as the tissue is frozen its structural integrity can be compromised by ice crystal formation [15]. Lyophilised AM can also be stored for long periods of time.

#### Preservation through heat or air drying

This process involved using heat or air to remove any residual moisture from processed tissue to preserve it [4]. This process prevents any damage from freezing the tissue. Literature has noted that AM preserved by heat or air drying are the thinnest tissues at around 20–30 microns [21]. This technique is relatively uncommon due to the difficulties in standardising the technique.

#### Low temperature vacuum evaporation

Low temperature vacuum evaporation techniques involve treating the tissue with a sugar protectant, such as trehalose to preserve and stabilise tissue structure, cellular membrane, and proteins [15, 22]. Residual water is delicately removed through cool temperatures paired with specified vacuums to lead to a dried product. The sugar protectants used in the preservation process replace intracellular water to prevent any damage to the AMs cellular matrix [15, 23]. Tissue preserved through low temperature vacuum evaporation can be shipped and stored without any logistical issues allowing it be available worldwide and in warzones [4].

### Irradiation methods

Most commonly tissue preparations are treated with antibiotic and antimycotic solution during their preservation processes to remove the remanence of bacteria and fungus, however, some tissue preparations undergo further sterilisation by irradiation such as gamma sterilisation [20] or by using fluids, such as carbon dioxide in a supercritical state [24]. However, there has been reports that terminal sterilisation can compromise tissue integrity [21, 22]. Recent publications by Marsit et al. [25] have reported an antibiotic-based, aseptic decontamination technique that can be used for dried AM.

### Amniotic membrane application methods

AM may be transplanted in several ways, dependent on the ophthalmic modality that is being treated [4, 13]. The type of transplantation required depends on the location, size and depth of the wound [4].

#### Inlay-graft (Graft)

An inlay-graft, also referred to as graft, application of AM is applied epithelial-side-up in the bed of the wound. The tissue acts as a barrier or scaffold, similar to the basement membrane that allows for epithelisation to take place on top of it [12] and therefore, the tissue is permanently incorporated into the ocular surface over time. A single or multi-layer graft may be placed, dependent on the depth of the wound. AM forms hemidesmosomes and desmosomes that provide its ability to incorporate into the ocular stroma [10].

#### Onlay-graft (Patch)

An onlay-graft, regularly referred to as a patch application of AM acts as a temporary biological bandage that acts as a barrier to protect the wound from external damage or pressure from the eyelids [4]. Patch transplantation is normally used in conditions with superficial damage or extreme inflammatory disorders. The amnion is applied epithelial-side-down against the wound [10]. For conditions that affect the entire mucosal surface, patch transplantation can protect from the formation of symblepharon and ankyloblepharon by providing a barrier between exposed or inflamed tissues [26, 27]. Patch AM transplantations can be replaced during the treatment period to give a renewed benefit and to help to drive ocular surface stabilisation [28].

#### Sandwich-graft (Sandwich)

The sandwich AM transplantation technique can also be referred to as the combined technique [10]. An inlay-graft is placed into the bed of the wound and an onlay-graft is used to cover it [4, 13, 29]. Sandwich graft technique has the purpose to guide the growth of a new epithelium between the inlay and onlay grafts [30, 31].

#### Folded AM graft

Eliezer et al. described the use of AM in trabeculectomy using a folded AM graft. A 5x5 mm graft is folded to have the stromal layer facing both superior and inferior sides of the implant. It is than fixated over the sclera with two 10.0 nylon suture [32].

#### In-office use

Most frequently, AM implant is used in procedures performed in theatres. However, sutureless in-office application has also been reported as a less invasive option for ocular surface diseases. The advantage of this method is that it allows the AM implant to be done faster and in earlier stages of ocular surface conditions, for instance keratitis, corneal ulcers, and chemical burns. Mcguaghy et all describe two different types of in-office AM implant manufactures: ProKera and AmbioDisk. Both are applied under topical antithetic and require saline irrigation of the graft. AmbioDisk is advised to be used with a bandage contact lens covering the AM disc [33].

## The benefits of amniotic membrane transplantation

The specific mode of action of AM has not been completely quantified but it is known to be a combination of the barrier function of the tissue from structural benefits, which is complimented by the bioactive proteins within the tissue structure [4, 13, 34]. These properties work synergistically to have a homologous benefit when used as a tissue transplant.

### Promotion of epithelisation

AMs ability to promote epithelisation is a dual synergistic effect between structural substrate benefits and the presence of bioactive proteins, which are delivered to the wound [4, 35, 36]. The extracellular matrix of the AM resembles the structure of the cornea and conjunctiva [4]. This allows the tissue to act as a replacement basement membrane in the case where tissue is lost or damage to provide a barrier for wound healing to occur on [30, 37]. AM contains a range of trophic factors, such as epidermal growth factor, neurotrophic substances and keratocyte growth factors that are delivered to the wound following application [35, 36], which have been shown to aid the healing process.

### Anti-inflammatory and immunosuppressive action

AM transplantation limits the development and continuation of the inflammatory process through an immunosuppressive mechanism that limits the infiltration of infiltrating immune cells. Shimmura et al. noted that inflammatory cells are trapped with the AM’s stroma, where they undergo apoptosis and therefore, do no cause an inflammatory cascade at the ocular surface [38]. AM has an extracellular matrix within the stroma, this complex is reported to contain heavy chain protease inhibitors of heavy chain 1 of inter-$\alpha$-trypsin and hyaluronan/pentraxin3 (HC-HA/PTX3) [39, 40]. The inhibition of HC-HA/PTX3 is known to contribute to the anti-inflammatory, anti-fibrotic and anti-angiogenic mode of action of AM. Finally, pro-inflammatory cytokines, such as interleukin (IL-)-1a, IL-2, IL-8, IL-10 and tumour necrosis factor-beta have their expression inhibited by AM transplantations [41].

### Mechanical properties

The AM tends to be between $20 \,{\rm and} \,500 \,\mu {\rm m}$ [9]. The basement membrane of the AM closely resembles that of the cornea and conjunctiva, regarding its collagen composition [42]. This property means AM can be an effective substrate to replace the missing ocular tissue to provide a basement membrane for epithelisation to take place [10].

## Glaucoma

For glaucoma patients with appropriate indication, AM can be transplanted as a secondary adjunct to surgical procedures to replace lost or missing conjunctiva, which can leave the sclera exposed [4]. AM is known to reduce scarring at the time of the filtering surgery; repair any early or late blebs and act as a protective cover in valve procedures [43]. When used in conjunction with any glaucoma surgery, AM transplantation is used to protect and cover any exposed sclera to improve both patient outcomes and the rate of complications [44, 45]. It should be noted that as AM is a human tissue, it can have variable persistence time dependent on the inflammation present at the ocular surface and the method of application. Although literature reports its high tensile strength and ability to be excellent for host-tissue integration [46]. Table 1 summarises some useful papers on glaucoma.

**Table 1:** Summary table: Amniotic Membrane (AM) in Glaucoma papers*

Author Year	Study type	Purpose	N (eyes)	Follow-up	Result
Ainsworth [63]	Series of 3 cases	AM on tube exposure of glaucoma tube shunt	3	6 months to 2.5 years	Successful re-epithelialization in all 3 cases
Amini [64]	Prospective clinical trial	AM covering Ahmend glaucoma valve implantation	7	16.8 ± 4.6 months	Suggests to be a safe and effective procedure. Likely to reduce the risk of bleb encapsulation
Budenz [58]	Prospective clinical trial	Compare AM with conjunctival advancement for glaucoma bleb leaks	2 groups 15 each	19 months	Survival Rate AM = 46%, Conjunctival adv. = 100%
Rauscher [59]	Randomized controlled trial	AM for leaking glaucoma filtering blebs vs conjunctival advancement	30	80 months	AM was more susceptible to early re-leakage, but can be an alternative to conjunctival advancement
Sheha [48]	Prospective randomized study	Compare trabeculotomy with mitomycin C with vs without AM transplantation	37 19 with mmc and AM; 18 with mmc alone	12 months	mmc and AM has lower IOPs and less complications rates than mmc alone
Sheha [3]	Retrospective case series	Evaluation of AM graft in glaucoma shunt surgery	44	22 ± 3 months	AM graft is a good tectonic support and allows visualisation of the underlying tube
Shen [2]	Systematic Review	Evaluate the safety and effectiveness of trabeculectomy with or without AM transplantation	Varied 67 articles	Varied	Association of AM transplantation shown to be more effective and safer than without it
Yazdani [53]	Double-blind Clinical trial	AM transplantation vs Mitomycin C for Ahmed glaucoma Valve	75	12 months	No statistically significant difference

*The benefits of AM in cornea and ocular surface are already well documented in the literature [6–8]

### In conjunction with trabeculectomy

For glaucoma surgeries, the use of AM transplantation in conjunction with trabeculectomy procedures is the most documented glaucoma indication [4, 13]. First reported by Fujishima et al. [47] the surgical procedure for AMtransplantation following trabeculectomy is yet to be standardised. Some studies report the placement of the AM below of scleral flap in a ‘inlay-graft’ fashion [48, 49], however, other studies state that the tissue is placed over the wound in an ‘onlay-graft’ fashion to over the entire exposed scleral bed [32, 44, 50]. As a whole, data shows the application of AM epithelial-side-down [2] when placed onto the wound. Although these differences are present, no obvious differences have been noted regarding outcomes dependent on surgical technique. Future research in this area has the potential to define if different surgical methodologies may lead to higher success rates.

In 2015, Wang et al. [51] conducted a meta-analysis on the data of device modified trabeculectomy procedures for glaucoma conditions. This analysis studied 33 papers that met the inclusion criteria, which included 18 studies utilising AM. These studies reported that tissue transplantation is associated with a lower intraocular pressure at 12 months, compared to trabeculectomy alone [51]. Although this review was limited by the loss of follow-up in 11/18 studies, but this collation of data presented the benefits of AM as an adjunct to this procedure.

Following this, Shen et al. [2] ran a meta-analysis of randomised controlled clinical trials that assess the AM assisted trabeculectomy to trabeculectomy alone. Shen et al’s meta-analysis included 174 eyes across 5 studies, the data noted that there was a statistically significant benefit in the intraocular pressure of patients in the AM treatment group at 3 months ($p < 0.0003$) and 12 months ($p = 0.02$). Whilst the complete success of patients’ procedures in the AM group were also significant at 6 months ($p = 0.02$) and 12 months ($p = 0.003$) compared to the trabeculectomy alone group. Additional to this, fewer complications were noted in the AM group. However, no benefit observed differences regarding the number of anti-glaucoma medications; hypotony; encapsulated bleb and choroidal detachment. In conclusion, Shen et al. noted that AM transplantation with trabeculectomy is a safe and efficient treatment compared to trabeculectomy alone.

Studies have researched the use of AM transplantation paired with mitomycin-c application when undergoing trabeculectomy to trabeculectomy and mitomycin-c alone. Sheha et al. [48] studied 37 eyes in a two-arm comparative study. Complete success at 6 months was noted 93.6% (15/16) of the study eyes, compared to 9/15 (60%) of the trabeculectomy alone control eyes. The AM treatment group saw statistically significantly improved intraocular pressure compared to the control group at 3-months ($p = 0.0002$), 6-months ($p < 0.0001$), 9-months ($p < 0.0001$) and 12-months($p < 0.0001$). Sheha et al. [48] noted that the use of mitomycin-c and AM combined in a trabeculectomy with mitomycin-c procedure providers lower intraocular pressure, higher success rates and fewer complications.

Most recently, Roque et al. presented a retrospective study of 51 eyes of 45 glaucoma patients, which compared trabeculectomy with mitomycin-c application to patients who had undergone trabeculectomy with mitomycin-c and amniotic membrane. This study showed no significant difference between the two groups, however, it should be noted that this was not a randomised and controlled cohort of patients due to the retrospective nature of the study [52].

During glaucoma procedures, mitomycin-c is used to limit scar formation and to improve the bleb formation following trabeculectomy procedures [53]. However, mitomycin-c can cause the formation of thin-walled blebs, which can lead to over-filtration, hypotony and can form serious complications such as: bleb leaks; bleb infections and endophthalmitis [53]. Therefore, AM has a potential to improve the success rate and lower the complications rate of trabeculectomies with mitomycin-c even further [2].

Published clinical data supports the use of AM in conjunction with trabeculectomy procedures, and this also may be supported using mitomycin-c in the procedure too. However, further research is required tos define the most beneficial surgical technique of above or below the scleral flap, as placement as an onlay-graft or inlay-graft.

Trabeculectomy surgeries is known to rely on the availability of mitomicin-C. At the end of 2019, the world experienced an international shortage of this vital medication for glaucoma surgeons [54, 55]. Taking this into account, studies regarding the AM as a possible substitute for mitomycin-C has an extreme importance to be considered.

### Leaking bleb fixture

For patients undergoing AM transplantation to manage a leaking bleb, the tissue is placed over the bare sclera, often epithelial-side-down [56]. The AM is supported by the corneoscleral limbus with the opposite side being undermined under the healthy conjunctival tissue and secured in place at both areas [56]. The aim of this treatment is to reduce inflammation, vascularisation and allow for the intraocular pressure of the patient to allow for stabilisation.

A meta-analysis was published by Bochmann et al., in 2012 [57] that noted the variations of treatments for managing late trabeculectomy bleb leaks. Within the Bochmann et al. analysis, one randomised controlled study of 30 eyes of 30 patients by Budenz et al. [58] matched the inclusion criteria and was included in the analysis. The study arms compared patients treated with conjunctival advancement to those who underwent AM transplantation. The primary outcome of the study was the sealing of the bleb leak confirmed by a negative Seidel’s test after 1 month, 1 year and 5 years. In the 19.5 ± 6.9 months follow-up period, the cumulative survival rate of the bleb reconstruction was 81% at 6 months and 74% at 1 year for the amniotic membrane group. However, it was at 100% for conjunctival advancement for the follow-up period. Recurrence of the bleb was seen in seven of the AM cases, with two persistent leaks and three that required repeat surgery. However, intra-ocular pressure, visual acuity and the number of glaucoma medications prescribed were comparable between the two groups.

The long-term outcomes ($73 \pm 7$ months) of the patient cohort included in the Budenz et al. study were evaluated by Rauscher et al. [59]. During this analysis, it was noted that there was no significant difference in patients’ intraocular pressure, final visual acuities, or difference in the number of glaucoma medications. Bleb vascularity was significantly lower in the AM group ($p = 0.2$) when comparing failed bleb repair, 7/30 of the AM group failed with 4/30 of the conjunctival advancement group failing ($p = 0.44$), showing no statistically significant difference. Rauscher et al. concluded that although prone to early potential failure, AM may be a suitable alternative to conjunctival advancement in the long-term.

Since the release of Bochmann et al’s meta-analysis, Sethi et al. [60] published a retrospective case series on patients who underwent sub-conjunctival AM draping to repair leaking cystic blebs. Of the 17 eyes of 16 patients included in the study at a follow-up of $21.4 \pm 7.3$ months, a success rate of 88% (15/17 eyes) was noted, patients saw a stabilisation of the intraocular pressure from $5.7 \pm 2.8$ mmHg to $13.1 \pm 3.4$ mmHg and LogMAR visual acuity improved from $0.7 \pm 0.8$ preoperatively to $0.1 \pm 0.1$ LogMAR units ($p < 0.030$).

The data regarding the use of AM transplantation as an adjuvant to manage leaking blebs is relatively mixed. Although the technique is viewed as being safe, there are more effective treatment methods available, such as conjunctival advancement. Therefore, this technique may be most viable in patients with a lack of conjunctival tissue, where alternative treatment options are required. Otherwise, further research is required to refine the surgical technique to evaluate if there is a potential for the improvement of AM use in the management of leaking blebs.

### Glaucoma valve or shunt implantation

A glaucoma shunt tube or valve will be covered using a range of transplantation materials. This includes: pericardium, donor corneal tissue, sclera, collagen matrixes or AM. The aim of covering the implantation is to avoid erosion, which can cause implantation exposure [61, 62]. Ainsworth et al. in 2006 [63] first noted the application of AM to manage exposed tubes following a glaucoma tube shunt surgery in a small case series of 3 patients. In one of these cases, they described a technique using a double AM graft to cover the tube from the glaucoma drainage device (GDD). The GDD tube is first covered by a full thickness scleral patch, sutured with four 9.0 nylon stitches. Then the first AM graft is placed with epithelial side up. The size of this graft is described to be larger than the above conjunctival defect. The conjunctiva is placed above the first AM implant, noticing that between the conjunctiva edges, a gap is kept. Four 10.0 nylon sutures fixate the conjunctiva to the underlying epsclera/AM draft. In sequence the second AM patch is applied over the epithelial defect. This second AM patch is larger than the epithelial defect and is placed with epithelial side down. Finally, four 6.0 nylon sutures are done at the margins of the second AM patch to keep it in the right position.

The use specifically in ahmed glaucoma valve procedure was noted by Amini et al. in 2010 [64]. The largest study in this area has been conducted by Yazdani et al. in 2016 [53], a randomised, double-masked 3 arm clinical trial of 75 eyes of 75 patients. The patient arms included ahmed glaucoma valve (AGV) alone compared to AGV with mitomycin-c compared to AGV with AM transplantation. Patients in the AM arm had their AGV wrapped with tissue prior to implantation with the aim to reduce fibrosis and postoperative vascularisation. Regarding intraocular pressure, at 12-weeks no statistically significant difference was noted ($p = 0.13$) between all three arms. Similar to this, there was no difference in the number of glaucoma medications taken by patients in any of the arms ($p = 0.22$). This study is limited by the short follow-up time of 12 months. A longer-term study is required to further assess the benefit of this treatment. Both AM and mitomycin-c have been shown to be safe methods of treatment, however, in this study, neither were associated with improved outcomes within the 12-month follow-up period.

In regard to glaucoma shunt addition, Sheha et al. in 2011 [3] conducted a retrospective case series of 44 eyes who underwent the implantation of a glaucoma drainage device with an AM graft. The patients had a follow-up of $22 \pm 3$ months. Of the cohort, 93% (41/44) of eyes had a successful procedure. The incidence of tube exposure was 2.3%, which is comparable to alternative substrate materials that are applied in these conditions. Sheha et al. [3] concluded that AM presented good tectonic support and allows for the clinician to have direct visualisation of the implanted shunt through the conjunctiva and tissue graft. Some cases have reported the use of a double layer AM transplantation when protecting a shunt tube to provide additional durability and support, if the AM transplantation were to begin to degrade [65].

The current body of literature with AM transplantation in glaucoma shunt or valve surgeries is relatively limited and offers the opportunity to be further explored to fully provide data regarding its long-term effectiveness for recipients. The most common reported surgical methodology for applying the AM in shunt procedures is during the primary procedure [66, 46], however, there is additional reports where AM has been transplanted following an exposed shunt tube, which may or may not have been covered with other transplant materials (i.e. scleral flap or pericardium) [67].

## Vitreoretinal procedures

The potential to utilise AM transplantation in retinal and vitreous procedures is a new, novel and developing area of research. AM has traditionally only been utilised for treatment for conditions of the ocular surface, but its wound healing function is now being placed in vitreoretinal procedures. AM has been proved a helpful tool in treatment of large macular tears, high myopic retinal detachment associated with macula hole, paravascular tears, serous macular detachment associated with optic pits, complicated retinal detachment and advanced age related macular degeneration (AMD) [68]. Table 2 summarises several important vitreoretinal papers.

**Table 2:** Summary table: Amniotic Membrane (AM) in Retina papers

Author Year	Study type	Purpose	N (eyes)	Follow-up	Result
Abouhusseinf [79]	Retrospective case of series	AM plugs for macular holes with hematogenous retinal detachment	14	6 months	All patients had macular hole closure
Caporossi [80]	Case report	New surgical technique using AM for retinal detachment due to paravascular retinal brakes in pathologic myopia	2	6 months	AM good option to treat posterior retinal breaks once internal limiting membrane has been previously peeled
Caporossi [75]	Retrospective interventional study	AM plug implants for failed macular hole closure	36	6 months	100% of closure
Caporossi [77]	Case series	AM plug for high myopic macular hole after previous membrane peeling surgery	16	12 months	All cases had some visual acuity recovery and 100% of closure
Garcin [69]	Prospective interventional case series	Large disc of trypan-blue stained lyophilized AM for complex macular holes treatment	10	12 months	Anatomic closer 80%, far vision improvement 90%, near vision improvement 60%
Rizzo [71]	Prospective interventional case series	AM for retina breaks repair and recurrent macular hole closure	14	4 to 12 months	Visual improvement in both groups. Promising surgical technique

### Macular hole closure

Recent publications have noted the use of amniotic membrane transplant in conjunction with vitrectomy for the treatment of macular holes [69–71]. Studies *in vivo* and *in vitro* have shown that AM transplantation can promote the regeneration of retinal tissue when placed under the fovea [72–74]. It has been noted that measuring the macular hole through ocular surface tomography, allows for the healthcare professional to alter the size of the AM graft specific to the defect [75].

Caporossi et al. report that in their experience, AM plugs are more precisely trimmed when using cutaneous punch. It is important to use an accurate size plug to achieve the best result. If the plug is smaller than the macular hole diameter, it will not develop the macular hole closure. Larger plugs may crinkle and stop the healing process.

The AM plug is implanted into the eye following the vitrectomy.

A 23-gauge trocar can be used if the plug size is smaller than 3 mm. However, if it is larger than 3 mm, a 20-gauge sclerotomy is indicated. In other to reduce the plug corrugation, a DSAEK- like technique has been described by Caporossi et al. This method uses a plastic support that holds the plug at the entrance of the trocar where it can be grabbed with a vitreoretinal forceps [68].

The AM plug is inserted under the neuroretinal margins of the hole [76] with the chorion layer facing the Retinal Pigment Epithelium (RPE) [68].

Garcin et al. in 2021 [69] noted how during a follow-up of 1-year that 10 eyes of 10 patients had shown to have successfully closed macular holes, which were previously refractory to the standard of care. Additional to this, Caporossi et al. in 2020 [77] studied the treatment of 16 eyes of 16 patients with high myopic macular holes of an axial length >30 mm. All patients were treated with an AM plug after undergoing a pars plana vitrectomy. Optical coherence tomography showed that 93.75% (15/16) of patients healed after two weeks, with the following patient having complete healing after a repeat application of the AM plug. The mean best correct visual acuity improved from 20/200 to 20/100 at the 6-month follow-up period. No adverse events were experienced during the treatment period [77]. Similar results have been noted in multiple studies, that showed an improvement in best correct visual acuity and complete healing of the macular hole at the 6-month period.

Caporossi et al. in 2021 [78] published a prospective, comparative study of 20 eyes of 20 patients all diagnosed with macular holes, which were failing to close. All macular holes were treated with an AM plug, with 10 eyes receiving transplantation with 20% sulphur hexafluoride tamponade and 10 eyes receiving transplantation with an air tamponade. Results showed that both groups had an improvement in their BCVA, and all macular holes closed at the 12-month follow-up appointment. However, this case reported that the use of air provided similar outcomes to the use of gas, an innovative technique.

The preliminary data regarding the use of human AM plugs to drive the healing of macular holes, which are recurrent or refractory to the standard of care is positive. Highlighting good healing rates within this group of patients. However, much of the data is only from select research groups and therefore, more expansive and comparative, randomised data is required to provide a complete conclusion. The potential to use air tamponade rather than gas tamponade presents another practice changing technique, which could benefit this group of patients.

### Retinal detachment

Whilst conducting research on macular hole closure, Rizzo et al. in 2019 [71] noted the use of AM transplant in patients with retinal breaks. Within the case series of 14 eyes of 14 patients, with 6 patients had retinal detachment (1 rhegmatogenous retinal detachment and 5 retinal detachment recurrences). All patients underwent transconjunctival pars plana vitrectomy with an AM plug being placed under the retinal tear. The BCVA for the cohort was $2.33 \pm 0.51$ logMAR pre-operatively and improved to $1.2 \pm 0.62$ logMAR post-operatively at the 3-month follow-up. No adverse events were experienced for any patients. The retina was shown to adhere over the AM plug in the retinal break site at 1-week post-surgery when using optical coherence tomography analysis [71].

Similar to Rizzo et al. in 2019 study multiple papers have supported the use of AM plugs in conjunction with vitrectomy for the treatment of retinal detachment [70, 79, 80]. Abouhussein et al. in 2020 [79] reported 14 eyes of 14 patients all diagnosed with rhegmatogenous retinal detachment co-existing with a macular hole. All the patients were treated with a human AM plug and had their condition improved from a BCVA of $1.87 \pm 0.31$ logMAR pre-operatively to $0.67 \pm 0.17$ logMAR post-operatively at the 6-month follow-up [79]. All the patients noted complete retinal attachment and macular hole closure at the 6-month follow-up appointment. This shows that all patients were effectively treated.

Innovation within this area is extremely new and upcoming and further research of larger patient numbers and randomised data is required to truly evaluate the benefit of AM transplantation in these conditions. However, it presents the growing scope regarding AM use and its capacity to be used in various ophthalmic modalities.

### Optic disc pit associated macular detachment

Optic disc pit may cause serous retinal detachment. The application of AM plugs has been a promising ally in this sight threatening condition. An AM plug placed inside the pit helps to improve the re-absorption of the intraretinal fluid. Enhancement of vision has been described at the 6^th^ month after the procedure. This is a faster result when compared with scleral autograft procedures, that has been reported to achieve improvement approximately 1 year later [68].

### Age related macular degeneration (AMD)

AM has been studied as a possible treatment for Geographic macula atrophy seen in late AMD patients. Caporossi et al. describe insertion of AM sheet in the macular area in eyes with AMD and macula atrophy. The implant is positioned with the chorion layer facing the RPE, and is performed after a previous surgical provoked retinal detachment. Visual improvement has been described from 20/2000 to 20/320, $p = 0.0084$.

## Risks of amniotic membrane use

During manufacture, precautions are taken during the donor selection and testing stages to protect any transplantation recipients, there is a minimal risk regarding the transplantation of human allogenic tissue as the risk of transmissible disease cannot be completely removed. AM is traditionally harvested by elective caesarean section to minimise the potential for transmission of disease or infectious agents during the delivery of the placental tissues [4]. All donors are tested for a range of transmissible diseases, such as HIV, Hepatitis, HTLV and more dependent on the country of collection and manufacturing body [13]. Processing of the AM can also be country dependent and a 6 month quarantine period is sometimes employed to reduce the risk of infection. After 6 months HIV tests are repeated to cover the immunological window period [13].

However, the risk of contamination cannot be completely removed, due to the low risk of the presence of infective agents, such as Variant Creutzfeldt-Jakob Disease (vCJD) and other emerging virus’, prions, or pathogens. Currently, there is no validated method for testing CJD infectivity of tissue donors that is routinely available for use [81].

## Conclusion

AM transplantation is a popular treatment methodology in ophthalmic care due to its immunosuppressive, anti-fibrotic and wound healing properties that are driven by the tissues potential to provide an environment or substrate for healing, dependent on the application method. Traditionally, AM transplantation has showed to be most utilised in corneal specialities, however, further research and development has highlighted its beneficial use as an adjunctive treatment in Glaucoma surgeries. However, the method of application of amniotic membrane in trabeculetomy is yet to be standardised.

For VR conditions, the data is only just emerging and although there is the lack a fully randomised clinical trials, it presents and exciting and innovative area of research and highlights the interest and beneficial perception of AM in ocular conditions of various specialities and aetiologies. AM transplantation has a large scope to be useful for long-term patient outcomes in conditions outside of corneal indications. Data is consistently emerging in these markets to provide well-rounded understanding of its benefits.
